# Hydrogel as a Biomaterial for Bone Tissue Engineering: A Review

**DOI:** 10.3390/nano10081511

**Published:** 2020-07-31

**Authors:** Shuai Yue, Hui He, Bin Li, Tao Hou

**Affiliations:** 1 College of Food Science and Technology, Huazhong Agricultural University, Wuhan 430070, China; yues@webmail.hzau.edu.cn (S.Y.); hehui@mail.hzau.edu.cn (H.H.); libinfood@mail.hzau.edu.cn (B.L.); 2 Key Laboratory of Environment Correlative Dietology, Huazhong Agricultural University, Ministry of Education, Wuhan 430070, China

**Keywords:** hydrogels, bone tissue engineering, mechanism, design, evaluation methods

## Abstract

Severe bone damage from diseases, including extensive trauma, fractures, and bone tumors, cannot self-heal, while traditional surgical treatment may bring side effects such as infection, inflammation, and pain. As a new biomaterial with controllable mechanical properties and biocompatibility, hydrogel is widely used in bone tissue engineering (BTE) as a scaffold for growth factor transport and cell adhesion. In order to make hydrogel more suitable for the local treatment of bone diseases, hydrogel preparation methods should be combined with synthetic materials with excellent properties and advanced technologies in different fields to better control drug release in time and orientation. It is necessary to establish a complete method to evaluate the hydrogel’s properties and biocompatibility with the human body. Moreover, establishment of standard animal models of bone defects helps in studying the therapeutic effect of hydrogels on bone repair, as well as to evaluate the safety and suitability of hydrogels. Thus, this review aims to systematically summarize current studies of hydrogels in BTE, including the mechanisms for promoting bone synthesis, design, and preparation; characterization and evaluation methods; as well as to explore future applications of hydrogels in BTE.

## 1. Introduction

**Figure 1:**
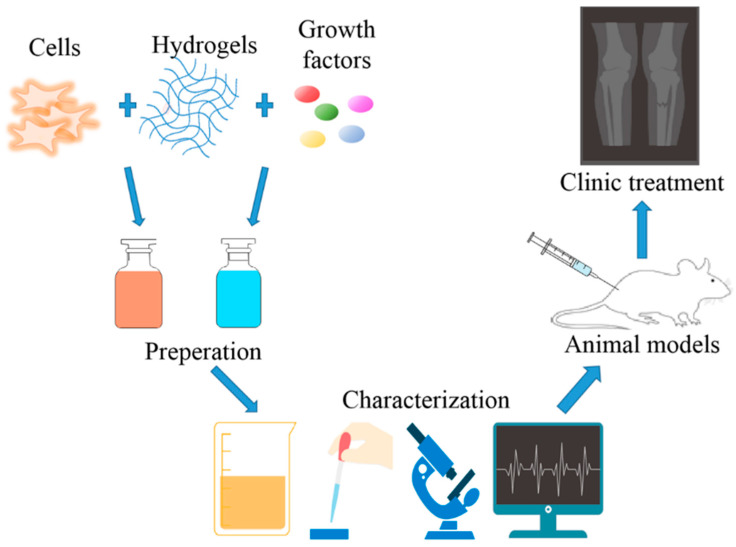
Hydrogel for bone tissue engineering research methods.

Bone is the second largest transplant tissue in the world. More than two million bone transplants are performed every year worldwide, while there is no satisfactory bone transplantation solution at present [1]. Traditional surgical treatments for fractures and bone defects mainly include bone grafts and metal prostheses, which have achieved good clinical results, but they also bring some serious disadvantages, such as infection, pain, high cost, and the need for additional surgery [2,3]. In some cases, such as bone defects, osteoporotic fractures, or bone defects/fractures in oncological patients, bone tissue regeneration after radiotherapy is hindered, which requires modern strategies such as bone tissue engineering (BTE) [4]. BTE has three elements—scaffolds, cells, and growth factors—combining engineering and cell science principles as shown in Figure 1 [5,6,7].

According to different chemical compositions, the biological materials commonly used as three-dimensional scaffolds are divided into metals, ceramics and glass-ceramics, natural and synthetic polymers, and composite materials [8]. The original biomaterials are usually selected from biologically inert substances that have the least toxicity in the human body, such as titanium or titanium alloys, stainless steel, cobalt chromium alloys, zirconia, carbon and other metals, ceramics, and synthetic polymers [9]. Later, because many natural biomaterials are important components of tissues and have good biocompatibility and biodegradability, synthetic and natural biodegradable polymers are also often used in biomaterials, such as collagen, calcium phosphate, and silica [10]. Nowadays, most biomaterials are designed to combine some active factors, hormones, and chemicals, and control the release to induce a good cellular response, such as inducing directional cell differentiation and improving cell survival. The ideal scaffold for BTE should have good biocompatibility, non-cytotoxicity and non-immunogenicity, and the carrier material should be suitable for cell or growth factor adhesion and transportation [11]. Compared with other biomaterials, hydrogels have similar porous structures to the extracellular matrix (ECM) and have good biocompatibility, thus they can be used as carrier materials for cells or bone growth to promote growth factors in BTE [12]. In addition, its soft texture can reduce the inflammatory response of surrounding cells and tissues, matching many soft biological tissues [12]. Thus, hydrogel is a suitable candidate.

After decades of development, more and more new hydrogels have been applied to BTE for targeted delivery of drugs and disease treatment. However, most of the studies are only in the experimental stage and have not been applied in clinical treatment; thus, the research on enhancing the safety and adaptability of hydrogels is what we should pay attention to in the future studies. In this review, research ideas for BTE hydrogels, covering mechanisms for promoting bone synthesis, material selection, preparation methods, characterization methods, and animal experiments, are summarized.

## 2. Hydrogels as Biomedical Materials for BTE

### 2.1. Compatibility with Organisms and Osteogenic Factors

For most fractures, bone tissue can heal itself, while complex fractures and diseases cannot heal themselves and require intervention to promote bone repair [13]. Several studies have shown that chondrocytes around the stromal site of bone injury have obvious chondrocyte death and metabolic dysfunction, and then extend from the injury site to the surrounding cartilage, thereby hindering the self-healing of bone tissue [13]. Various growth factors and cells are commonly used to promote bone formation in BTE as shown in Table 1, such as bone morphogenetic proteins (BMP-2–BMP-4 and BMP-7), insulin-like growth factor (IGF), fibroblast growth factor(FGF), vascular growth factor (VEGF), platelet-derived growth factor (PDGF), and mesenchymal stem cells (MSCs) [14,15].

Scaffold materials in bone tissue engineering can provide cells with a three-dimensional space for survival, which is beneficial for cells to obtain sufficient nutrients, perform gas exchange, eliminate waste, and regulate the morphology and function of tissue engineering cells. Scaffold materials can effectively support protein absorption and cell adhesion, so that the cells grow according to the prefabricated three-dimensional scaffold, and then this cell material composite is implanted into the bone defect site [16]. As the biological material is gradually degraded, the implanted osteocytes continue to proliferate to achieve the purpose of repairing bone defects. The influence of biomaterials on bone regeneration is mainly through the interaction between cells and surrounding biomaterials, in which interactions of cells play a main role in determining the behavior of cells on the surface of biomaterials [17]. Integrin is a heterodimeric receptor in the cell membrane, which acts as a linker between cells and substrates by binding to adhesion proteins on the surface of biological materials [18]. It is the key determinant of the subsequent cell activity including cell morphology, migration, proliferation, and differentiation. Therefore, the chemical composition, mechanical properties, hydrophilicity, and morphology of biomaterials are the key factors controlling the corresponding materials to control cell behavior. Many macromolecules similar in structure to the extracellular matrix, such as collagen, laminin, and fibronectin, are often used to prepare hydrogels to regulate the properties of cells attached to them. Arginine–glycine–aspartate (RGD) is one of the earliest studied cell adhesion peptides, originally derived from fibronectin, and has been widely used in the preparation of hydrogels to enhance cell adhesion [19]. Dosier et al. [20] demonstrated that the use of Arg–Gly–Asp-functionalized alginate hydrogel to deliver adult stem cells together with BMP-2 enhanced bone regeneration compared with acellular hydrogels containing BMP-2.

The ideal scaffold material should meet the following requirements; (1) good biocompatibility, non-immunogenicity, and non-toxicity; (2) degradable absorption and a degradation rate that can best match the formation of new bone; and (3) good porosity and three-dimensional structure, with a large surface volume ratio.

**Table 1:** Latest studies on hydrogels with cells and growth factors for bone tissue engineering: their preparation, methods of characterization, and application/evaluation.

Serial Number	Carrier Materials	Core Materials	Preparation	Characterization	Animal Models	Application	Year	References
1	Oxidized alginate-gelatin hydrogel	murine bone marrow stromal cell line	Covalently crosslinked	1. Porosity and Morphology; 2. In Vitro Apatite Formation and Degradation Behavior; 3. Cell Viability and Metabolic Activity.	-	Enhance the degree of osteogenic differentiation	2016	[21]
2	RGD-oxidized sodium alginate (RGD–OSA)–N-succinyl chitosan (N-SC) hydrogels	BMSCs	Schiff base reaction	1. FT-IR, SEM, mechanical properties, degradation experiments; 2. Cell viability, In vitro osteogenic and endothelial differentiation of rBMSCs.	-	RGD-grafted oxidized sodium alginate N-succinyl chitosan hydrogel might be an optimal material for bone tissue engineering scaffold whenever it is used alone, or composed with other materials.	2013	[22]
3	Nano-hydroxyapatite/glycol chitosan/hyaluronic acid composite hydrogel	-	Continuous ultrasound	1. FTIR, XRD, SEM, Porosity test, Swelling test; 2. In vitro degradation test, In vitro cytocompatibility test.	-	n-HA/G-CS/HyA composite hydrogels might be a promising candidate for bone tissue engineering applications.	2018	[23]
4	Cholesteryl group- and acryloyl group-bearing pullulan hydrogel	FGF-18 and BMP-2	Cross-linking with thiol-bearing polyethylene glycol	1. μCT Imaging, Histological examination, X-gal staining.	Calvarial defect model	The combination of the CHPOA/hydrogel system with the growth factors FGF18 and BMP2 might be a step towards efficient bone tissue engineering.	2012	[24]
5	An electrospun nanofiber mesh and alginate hydrogel	RhBMP-2	Carbodiimide chemistry	1. 2D radiographs and 3D in vivo μCT imaging, Torsional testing; 2. Histological analysis, Analysis of vascularity during bone regeneration.	Segmental defect model	This hybrid growth factor delivery system may be clinically useful for bone regeneration in the case of fracture non-unions and large bone defects.	2011	[25]
6	Gelatin-hydroxyphenylpropionic acid hydrogel (GHH)	TMSC	Enzyme-catalyzed	1. In vitro viability assay; 2. Biochemical analysis of blood samples, μCT Imaging, visceral fat mass measurements.	OVX mice	A more safe and effective therapy for osteoporosis.	2018	[26]
7	Carboxymethyl chitosan (CMCh) -amorphous calcium phosphate (ACP) hydrogel	BMP-9	PH-triggered self-assembled	1. SEM, TEM, DLS, FTIR, pH Responsiveness, Viscosity, and Injectability; 2. Cell Proliferation (WST-1) Assay, Gaussia Luciferase (GLuc) Assay, Cytotoxicity, Cell Proliferation, and Biocompatibility Analysis in cell experience; 3. μCT Imaging, HE Staining, and Trichrome Staining in mice model.	Athymic nude mice	The CMCh-ACP hydrogel itself is osteoinductive and induces the expression of osteoblastic regulators, while augmenting BMP9-induced osteogenic differentiation.	2019	[27]
8	Glyco-nucleo-lipids containing a fluorinated carbon chain (GNF)-collagen injectable hybrid hydrogel	HASCs	Thermo-gelation process	1. Physical and structural characterization of the composite hydrogels; 2. In vitro interactions between hydrogels and hASCs, in vitro adhesion and proliferation of hASCs; 3. In vitro and in vivo differentiation of hASCs-hydrogels complex.	Immuno-deficient mice	To promote hASCs differentiation towards the osteoblastic phenotype, and to elicit the formation of bone-like structures in an ectopic site in vivo.	2018	[28]

### 2.2. Osteoconductive Activity

Osteoconduction, the ability to form new bone on the surface of biomaterials, is a prerequisite for biomaterials to achieve functional bone regeneration. It is the bone conduction material that supports the migration, proliferation, differentiation of bone progenitor cells, vascular growth, and bone matrix deposition and calcification [29]. Osteoconductive materials do not provide bone progenitor cells, osteoblasts, or inducible factors. Instead, capillaries, perivascular tissues, and osteoblasts grow from host tissue into biomaterials, and eventually new bones are formed in porous structures [30]. In addition to hydrogels, biological or non-biological three-dimensional structure material that is biocompatible and in close contact with the host’s vascular bed and osteoblasts can also be used as a scaffold for the growth of new bone tissue, such as hydrogels porous ceramics and glass, porous plastics, porous metals, etc. [31].

Osteogenesis is formed by the proliferation, growth, and maturation of osteoprogenitor cells with a well-defined differentiation direction into osteoblasts. Hydrogels are in contact with the host bone’s internal and external periosteum, bone marrow, and other tissues with osteoblasts for osteogenesis. In the process of bone healing, the bone osteoconductive activity of hydrogel depends on its properties to some extent. [32]. Hydrogels with high porosity and large surface area have fast and safe bone conduction. In general, osteoconductive materials are inactive and provide space for fibrous vascular tissue to grow in as passive scaffolds. Many natural and non-biological materials used in preparing hydrogels are commonly used as bone graft substitutes for osteoconduction, which have the advantages of adjustable properties, good biocompatibility, and nonimmune response. Hydroxyapatite (HA) and tertiary calcium phosphate (TCP) have good bone conductivity because they are similar to natural bone minerals [33]. Type I collagen facilitates mineral deposition by binding to non-collagen matrix proteins, thereby initiating and controlling mineralization, and is also an osteoconductive material [34].

Bioglass can also be combined directly with bone for use in bone conductive materials. Although it is generally considered to have no bone conductivity, titanium oxide formed spontaneously from titanium exposed to air and water electrolytes is stable in the body and cannot be biodegraded [35]. Titanium dioxide, especially the anatase crystal structure, is beneficial to the formation of apatite and has excellent bone conduction capacity in the body [36]. The coating can be combined with the biomaterials in hydrogels, such as poly (lactic acid) (PLA) and poly(ε-caprolactone) (PCL) to increase the bone conduction capacity of composite materials. Studies have shown that the gelatin–chitosan nanocomposite membrane synthesized by ultraviolet irradiation, including hydroxyapatite and titanium dioxide nanoparticles, has good biodegradability, biocompatibility, and bone conductivity, and can be used as a good substitute for bone regeneration membrane, with high potential in orthopedic applications [36].

### 2.3. Osteoinductive Activity

**Figure 2:**
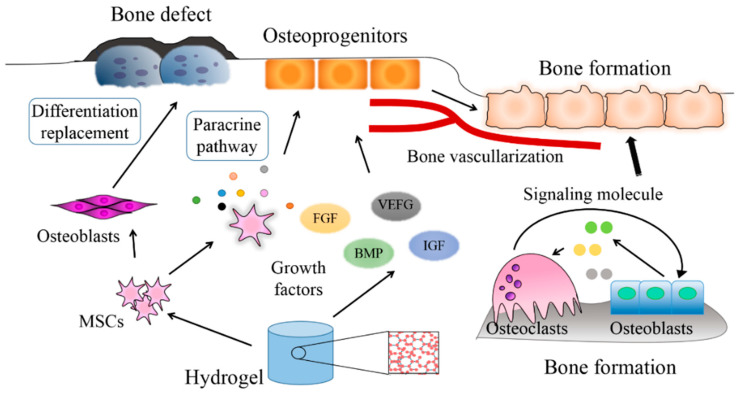
The mechanisms of mesenchymal stem cells (MSCs) and growth factors in tissue damage repair: differentiation replacement and paracrine pathway. (MSCs: marrow mesenchymal stem cells; BMP: bone morphogenetic proteins; IGF: insulin-like growth factor; FGF: fibroblast growth factor; VEGF: vascular growth factor.)

Osteoinductive materials can adsorb endogenous growth factors such as BMPs, induce mesenchymal cells to chemotaxis and migration into the material, and form bone tissue [37]. To investigate whether a biomedical material has bone induction, it is usually tested by inserting it into an ectopic soft tissue other than bone, such as muscle, myofilm, or subcutaneous, and by observing the formation of new bone tissue in or around the material. The osteoinductive properties of biomaterials are mainly affected by two pathways: differentiation replacement and paracrine pathway [14,15] (Figure 2). Biomaterials are highly attractive for the osteoinductive proteins present in these injury sites, and local enrichment of growth factors can promote bone formation at the injury sites [38]. The gathered bone morphogenetic proteins can stimulate chain-level reactions in cells, induce mesenchymal cells to differentiate into chondrocytes, secrete the cartilage matrix, and then mineralize to form bone. On the other hand, the osteoinductive properties of biomaterials are partly due to the promotion of blood vessel formation in the materials. Angiogenic factors such as transforming growth factor and insulin-like growth factor, which are released from hematomas, fractured ends, and inflammatory cells, recruit osteoblasts and promote their differentiation, thereby continuously generating new bone. Mesenchymal stem cells tend to grow in an agglomerated form on the surface of hydroxyapatite, which is a commonly used osteoinductive material for hydrogel preparation. Natural and synthetic hydroxyapatite can mediate cell proliferation, differentiation, and mineralization through MEK1/2-ERK1/2 and JNK MAPK pathways [39].

Additional growth factors and cells can be transmitted through the hydrogel when the ability of self-recovery is insufficient, which can promote the proliferation and differentiation of mesenchymal stem cells and have a better effect on promoting bone synthesis. However, inflammatory response, lipogenesis, difficulty in dose control, and long-term retention of supplemental growth factors in the injured area may affect its function and even be harmful to the human body, requiring the hydrogel to act as a slow-release agent [40,41]. Yoon et al. [42] prepared an injectable glycol chitosan hydrogel system containing BMP-2 and transforming growth factor-β1 (TGF-1) for in vitro and in vivo scaffolds of bone formation, which can be released continuously for 30 days to increase bone volume and bone density at bone defect sites.

## 3. Design of Hydrogels for Bone Tissue Engineering

**Figure 3:**
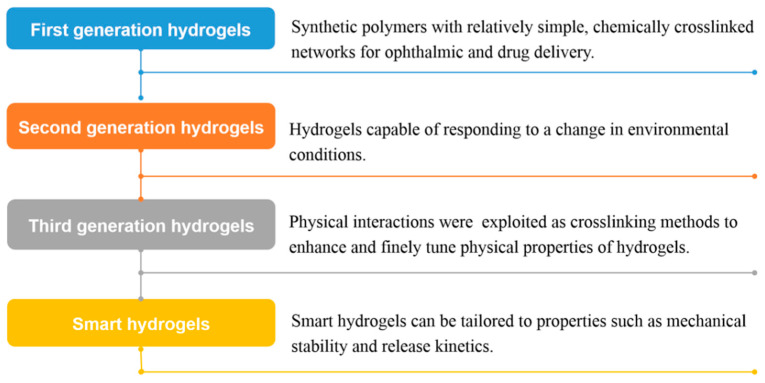
Development stage of hydrogels.

The development of hydrogels has gone through four generations as shown in Figure 3. Wichterle and Lim first described ethylene glycol monomethacrylate porous polymers with adjustable mechanical properties and water content in 1960, and they were successfully applied to contact lenses [43]. Then, studies on hydrogels shifted from a simple chemical single polymer network, such as poly(vinyl alcohol) (PVA), poly(ethylene glycol) (PEG), and poly(2-hydroxyethyl methacrylate) (pHEMA) [12], to the second generation hydrogels, stimulus-responsive hydrogels, as well as in situ hydrogel [44]. Nalbandian et al. [45] prepared pluronic hydrogel which can be used as artificial skin for the treatment of full thickness thermal burns and control the release of antimicrobial silver nitrate or silver lactate. In addition to hydrophobic interactions, physical interactions such as stereocomplexation and inclusion complexation were then used as cross-linking methods in the third-generation hydrogels, to provide the possibility of improving and fine-tuning the release performance [12]. Nostrum et al. [46] prepared a chiral opposite oligo(lactic acid) side chain stereocomplex hydrogel with phosphonanted hydrolytic polymaleic anhydride, which has a significantly longer degradation time than the dextran stereployocomplex hydrogel. Currently, organic and polymer chemistry and nanotechnology have been applied to the study of hydrogels, giving the fourth-generation hydrogels an unprecedented structure and new properties, meaning they can be used for more accurate targeted delivery [47].

### 3.1. Sources of Hydrogel Materials

**Figure 4:**
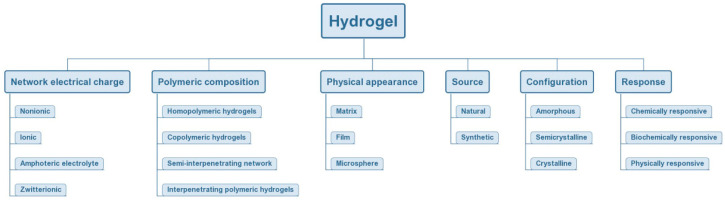
Classification of hydrogels based on the different properties.

Hydrogels can be classified according to their different properties, such as sources, polymeric composition, type of cross-linking, physical properties, biodegradation rates, network electrical charge, and response to environmental conditions (Figure 4) [48,49].

According to the source of hydrogel materials, they can be divided into natural materials and synthetic materials [50]. Natural materials are directly obtained from natural resources, and synthetic materials are prepared through chemical reactions. They generally have good biocompatibility and biodegradability, and most of these polymers are water-soluble [51]. Hydrophilic surfaces allow cells to easily adhere, proliferate, and differentiate, but the mechanical properties and stability of natural biomaterials are poor [52]. Gelatin [53], hyaluronic acid [54], alginate [25], chitosan, dextran [55], etc. are natural materials commonly used to prepare hydrogels [56,57,58]. Naoki Sasaki et al. [59] demonstrated that gelatin hydrogels containing basic fibroblast growth factor (bFGF) can promote the healing of proximal sesamoid fractures, in which hydrogels are safe for the injured site and the degradation can be controlled by controlling the degree of cross-linking. Synthetic biomaterials have the advantage of being controllable and reproducible [60]. However, the biocompatibility and material safety of synthetic biomaterials are poor, and as compared with natural biomaterials, synthetic biomaterials have lower biological activity [61]. Common synthetic biomaterials include PEG [52], poly (N-isopropylacrylamide) (PNIPAAm) [62], polycaprolactone (PCL) [63], poly(L-glutamic acid) (PGA) [64], polypropylene fiber (PPF) [65], and PVA [66]. For example, PEG is a promising hydrophilic biomaterial for BTE, enabling bind to hydrophobic but degradable polymers such as polylactide (PLA) and PCL to create an amphiphilic polymer [67]. Fu et al. [24] studied the synthesis of triblock PEG-PCL-PEG copolymer (PECE) copolymers, collagen, and nano-hydroxyapatite hydrogel composites, combining the advantages of PECE hydrogels, collagen, and n-HA fillers with injectability and thermal sensitivity. This hydrogel composite has good bone regeneration ability compared with the self-repairing process.

According to the mechanism of cross-linking, hydrogels are divided into four categories: homopolymeric hydrogels, copolymeric hydrogels, semi-interpenetrating network (semi-IPN), and interpenetrating polymeric hydrogels (IPN) [48,68]. The cross-linking network of homopolymer hydrogels is formed by polymerization of a single hydrophilic monomer [69], whereas the copolymeric hydrogels consist of two or more different monomer units, which have at least one hydrophilic component to prevent swelling [70]. Polymers are classified as semi-IPN when one linear polymer penetrates another crosslinked network without any other chemical bond between them [71]. IPN is often formed by binding two polymers together with immersing prepolymerized hydrogels in a solution of monomer and polymerization initiator [70]. In BTE, semi-IPN can more effectively maintain a fast dynamic response rate to pH or temperature with its unrestricted interpenetrating elastic network, and the main advantage of IPN is that they can produce relatively dense hydrogel matrices with higher mechanical properties, more efficient drug loading, and controlled physical properties [70,72,73]. Zhang et al. prepared a biodegradable hybrid double-network hydrogel (DN) by interspersing a methacrylated gelatin (GelMA) network into a well-defined nanocomposite (NC) hydrogel consisting of methacrylated chitosan (CSMA) and polyhedral oligomeric silsesquioxane (POSS) via a two-step photo-crosslinking process [74]. It was found that the DN hydrogel reached a much higher compression stress of nearly 2.0 MPa at a strain of 89%, while the pristine NC hydrogel was only able to withstand 0.3 MPa compression stress at 78% strain. For even more challenging tensile tests, the DN hydrogel achieved a high fracture stress of 131.1 kPa, which was more than 11 and 4 times stronger than that of methacrylated gelatin and NC hydrogels (11.5 and 32.4 kPa), respectively [74]. Therefore, DN hydrogels showed excellent stiffness and toughness, better than gel and NC hydrogels, indicating that the synergistic effect of the two networks entangled with each other could withstand greater external forces.

Hydrogels that can respond to external stimuli are known as stimulation-responsive hydrogels, whose volume, network structure, mechanical strength, and other properties change with the stimuli. Hydrogels can be divided into physical response hydrogels, chemical response hydrogels, and biochemical response hydrogels according to the types of external stimuli to which they can respond. Physical stimuli include temperature, pressure, light, electric fields, and magnetic fields, while chemical stimuli include pH, ionic strength, and chemical agents. Biochemical stimuli include reactions to ligands, enzymes, antigens, and other biochemical drugs [48]. In BTE, temperature-responsive hydrogels are commonly used as injectable hydrogels, which are liquid at room temperature and can be rapidly gelled at physiological temperature at specific local tissues [75,76]. Injectable hydrogels can reach the defect site by minimally invasive surgery, can treat any shape of deformity, and can also be used for drug delivery and fixation of injured bone tissue. Hydrogel is mainly used as a scaffold in BTE. It is a basic subunit that provides mechanical strength; a site for cell attachment, proliferation, and differentiation; and a carrier for protection and delivery of growth factors [77].

### 3.2. Synthesis of Hydrogel

**Figure 5:**
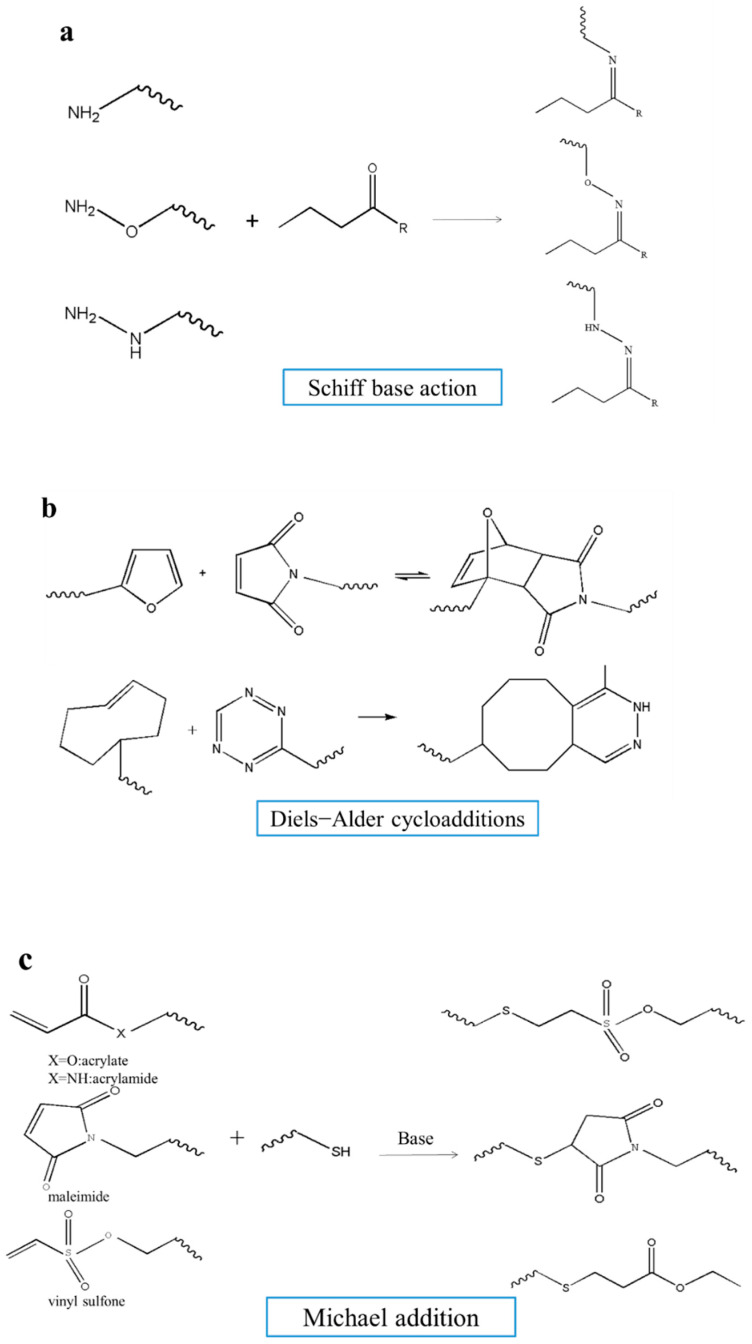

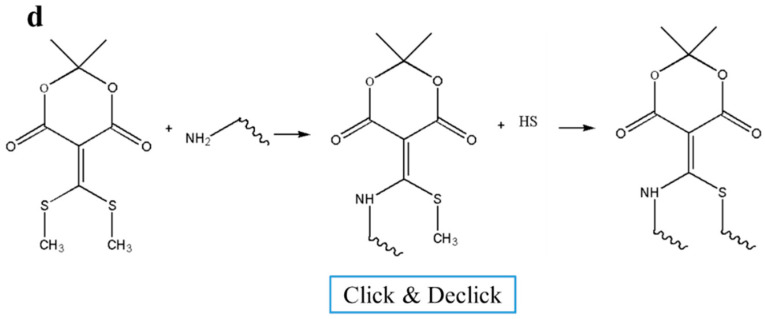
Chemical crosslinking methods to form hydrogels. (Chemical hydrogels are mainly connected by (**a**) Schiff base reaction, (**b**) Diels−Alder cycloadditions, (**c**) Michael addition reactions, (**d**) click chemistry).

**Figure 6:**
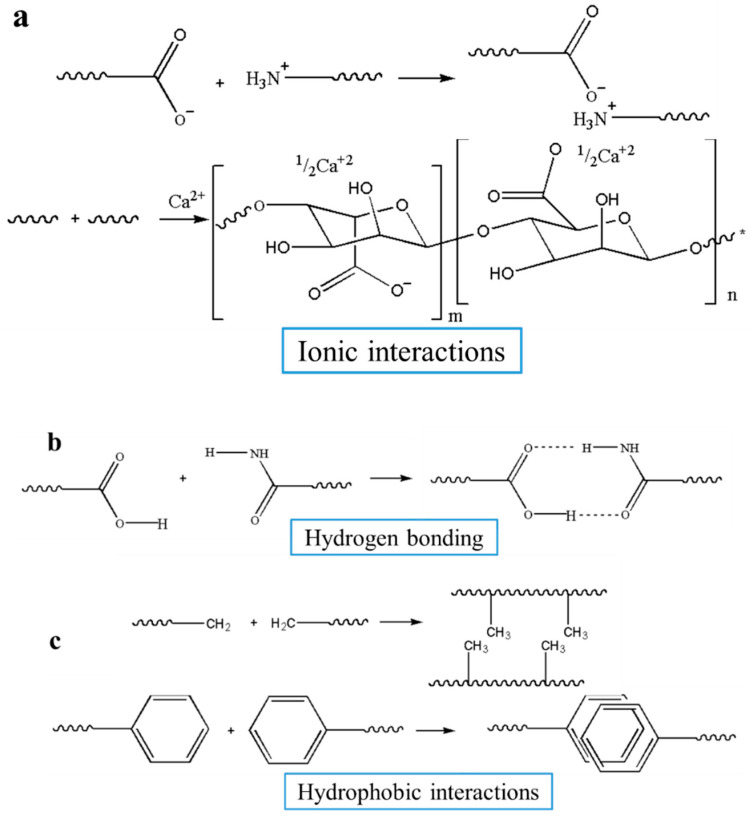
Physical crosslinking methods to form hydrogels. (Physical hydrogels are mainly connected by (**a**) ionic interactions, (**b**) hydrophobic bonding, (**c**) hydrogen interactions).

The network of hydrogels is mainly prepared in two ways: chemical cross-linking (Figure 5) and physical crosslinking (Figure 6). Physical hydrogels are connected by ionic interactions, electrostatic interactions, hydrophobic interactions, crystallization, and hydrogen bonding [78,79]. Different methods to synthesize chemically crosslinked hydrogels include Michael addition reactions, Schiff base reaction, Diels−Alder cycloadditions, free radical polymerization, and other click chemistry [79].

Physical noncovalent bonding mechanisms cause macromolecules to fold into scaffolds possessing well-defined structures and functionality [80]. Physical hydrogels are increasingly valued by researchers because they do not require crosslinkers and can be self-assembled under specific conditions. It has now been reported that a wide variety of physical injectable hydrogels can be formed without any chemical irritation, such as ion-sensitive and stress-sensitive hydrogels [56]. Ionic interactions are commonly used in the preparation of natural polysaccharide hydrogels, such as calcium silicate/sodium alginate composite hydrogels [81]. It can effectively support the adhesion, proliferation, and differentiation of osteoblasts and angiogenic cells, and has broad application prospects in bone regeneration and tissue engineering applications [81]. Hou et al. [82] developed an injectable self-assembling hydrogel connected by hydrogen bond, which is biocompatible, biodegradable, and capable of sustainable release of biomolecules, and can be combined with different types of cells and biomolecules for BTE.

Compared with physical methods, chemical methods can greatly improve the control of the flexibility and spatiotemporal accuracy of the crosslinking process, which is better at stabilizing the hydrogel matrix [80]. The Michael addition reaction can be carried out in aqueous media, at physiological pH, and at room temperature, which is a promising strategy for preparing biomimetic hydrogels [83]. However, the high-efficiency reaction with the acrylate Michael acceptors has high cytotoxicity, which can be overcome to some extent by shortening the reaction time [79]. Under physiological conditions, the Schiff base reaction between an aldehyde group and amine can form a non-toxic gel with good biocompatibility in a short time, which provides a simple and reliable method for the formation of cell-friendly materials [84]. Due to the dynamic equilibrium of Schiff base bonds, Schiff base reactions are also used to prepare self-healing hydrogels [85]. Click chemistry is being studied and applied to produce new, promising materials with interesting properties for encapsulation of cells and growth factors [86].

The past decade has witnessed an improvement in hydrogel preparation in the direction of combining various components and mechanisms with improved hydrogel properties, which typically exhibit excellent physicochemical properties, thereby enabling hydrogels to be cutting-edge biomaterials and looking at how these materials will eventually translate into clinical applications [80]. Hydrogel scaffolds can be used as a substitute for extracellular matrices, where cells can grow and proliferate, drugs can be released, and nutrients can be diffused [87]. Hydrogels can be prepared into any shape and size according to the application [88,89]. They can be cast or formed into practically any shape and size according to the application, and are divided into macroscopic hydrogels, microgels [90], and nanogels [91] by size. Many new technologies have been used to prepare hydrogel particles of different sizes, including microfluidics methods, emulsion, and nano molding techniques, where the size of the hydrogel particles can be controlled by controlling the gelation conditions or processing parameters [92]. The mesh size [93] and the mechanism of cross-linking of hydrogels determine how the drug diffuses through the hydrogel because they control the steric interactions between the drugs and the polymer network, which can be controlled by the concentration of polymer and crosslinker as well as external stimuli such as temperature and pH [91,94]. For example, semi-IPN [95] can more effectively maintain a fast dynamic response rate to pH or temperature with its unrestricted interpenetrating elastic network, and the main advantages of IPN [95] is that they can produce relatively dense hydrogel matrices with higher mechanical properties, more efficient drug loading, and controlled physical properties. In addition, the bioadhesive properties of hydrogels is also an important factor in application, because hydrogels with good adhesion can prolong the retention time of embedded drugs in the target site. Some polymers, such as chitosan and poly (acrylic acid), have been found to be mucoadhesive. Stimulation-responsive hydrogels that can respond to external stimuli are also a hot topic in biomaterials. The volume, network structure, mechanical strength, and other properties will change with the stimuli. Hydrogels can be divided into physical response hydrogels, chemical response hydrogels, and biochemical response hydrogels according to the types of external stimuli to which they can respond. Physical stimuli include temperature, pressure, light, electric fields, and magnetic fields, while chemical stimuli include pH, ionic strength, and chemical agents. Biochemical stimuli include reactions to ligands, enzymes, antigens, and other biochemical drugs. In BTE, temperature-responsive hydrogels are commonly used as injectable hydrogels, which are liquid at room temperature and can be rapidly gelled at physiological temperature at specific local tissues [75,76]. Injectable hydrogels can reach the defect site by minimally invasive surgery, treat any shape of deformity, and be used for drug delivery and fixation of injured bone tissue. More and more new chemical strategies and control principles with increasing adoption of interdisciplinary approaches will be applied to the preparation of hydrogels to develop a wide range of useful systems that provide reliable stimuli and release on demand at high levels of control [96].

### 3.3. Characterization of Hydrogel with Physicochemical Properties

**Figure 7:**
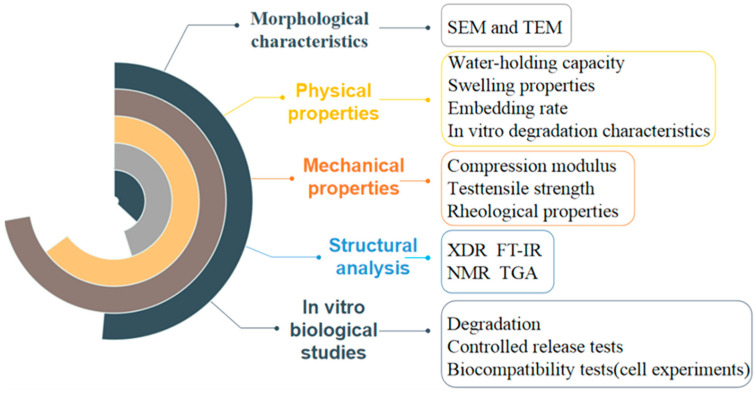
Summary of characterization methods for hydrogels in bone tissue engineering applications.

A key step in the application of hydrogel scaffolds to BTE is the quantitative and qualitative characterization of hydrogel properties, which allows the hydrogel to be tailored to specific application requirements [97]. In the study of hydrogels, different methods are commonly used to characterize hydrogel materials (Figure 7).

#### 3.3.1. Morphological, Physical, Mechanical, and Structural Analysis

The size of the meshes between polymer molecules in the hydrogel network can vary from macropores of 10–500 μm to pores of 5–100 nm, which are designed for inducing regeneration [92]. Scanning electron microscopy (SEM) and transmission electron microscopy (TEM) are commonly used to observe the surface morphology and internal porous structure of hydrogels and other biomaterials. The obvious difference between the two devices is the optimal spatial resolution they can achieve. For example, the photopolymerizable composite hydrogels were characterized by SEM and TEM, which provides strong evidence for hydrogel-carrying osteogenic synthesis factors as a substitute for BTE. SEM showed that hydrogels with a porous microstructure and pore wall were smooth. The internal structures of the hydrogel can be observed through TEM, and its resolution can reach 0.2 nm [98].

The physical properties of hydrogels are considered to be one of the most important factors in controlling bone regeneration [99]. Physical properties usually include water-holding capacity, swelling properties, and embedding rate of hydrogels. The water-holding capacity of hydrogel samples was determined through a centrifugation method [100]. The encapsulation efficiency can be measured by different materials such as spectrophotometer or high-performance liquid chromatography (HPLC) according to the different properties of the entrapped drug, which is also an important physical property of hydrogel [101].

As BTE applications require hydrogels with different properties, it is important to characterize the mechanical properties of hydrogels made from different materials and preparation methods. Some hydrogels used for drug sustained-release need to be biodegradable, and some hydrogels that need to have similar properties to extracellular matrix (ECM)can be used as 3D cell scaffolds to be implanted into the skin of animal models. Thus, different measurement methods, such as compression modulus test and tensile strength, and rheological properties are used to evaluate the mechanical properties of hydrogels.

As the presence of different functional groups and chemical bonds has an important influence on the properties of hydrogel, it is necessary to analyze the presence of different functional groups and linkages in the newly prepared hydrogel. X-ray diffraction (XDR), Fourier transform infrared spectroscopy (FT-IR), nuclear magnetic resonance (NMR), and thermogravimetric analysis (TGA) are usually used to analyze the hydrogel structures.

#### 3.3.2. Degradation and Controlled Release

In vitro degradation and controlled release tests are often performed on hydrogels for different applications such as subcutaneous injection or transplantation of hydrogels. For the degradation experiment, hydrogel material is placed in phosphate-buffered saline (PBS) and incubated for 37 °C for a certain period of time [102]. Similarly, in vitro release of drugs was analyzed by incubating a drug-embedded hydrogel in PBS at 37 °C for varying lengths of time, and the ratio of the cumulative release was calculated based on the total amount of drugs obtained from the initial weight of the hydrogel [103].

When hydrogels are used for transdermal drug delivery, skin permeability was investigated using the Franz diffusion cell system [104]. The skin removed from the abdomen of nude mice and normal mice was placed between the donor and acceptor chambers, with the stratum corneum facing the donor chamber. The material to be tested is placed on the skin in the donor chamber, the PBS is added to the receptor chamber, and the donor chamber is sampled at a set time to quantitatively determine the amount of drug permeated. The tested material was deposited on the skin in the donor chamber, and PBS was added to the receptor chamber. For the qualification assay, the amount of drug penetration is determined using the sample taken from the donor chamber at a set time [105].

## 4. Biological Properties of Hydrogel for Medical Application

### 4.1. Evaluation of Biocompatibility

In BTE, hydrogel materials are commonly used as a scaffold for cells. The safety of materials on embedded cells and implant sites is necessary. In vitro biocompatibility tests are usually carried out by cell experiments to observe the survival of cells in hydrogel materials through a series of indicators. The morphology of cells in a hydrogel scaffold can be observed by an inverted microscope. Cell growth and proliferation can be quantified by MTT (tetrazolium salt, 3-[4,5-dimethylthiazol-2-yl]-2,5-diphenyl tetrazolium bromide) assay. AO/PI (acridine orange/propidium iodide) staining, DAPI (4,6-diamidino-2-phenylindole) staining, and resazurin assay are also useful methods [101]. Cell growth was also evaluated at protein levels by analysis of the biosynthesis of the ECM, stained with toluidine blue dye, and observed under an inverted microscope [106]. Additionally, alkaline phosphatase (ALP) is an obvious feature of osteoblast differentiation and one of the most common indicators of hypertrophic differentiation [107].

### 4.2. In Vivo Biological Studies

Hydrogel development is mainly used in the treatment of bone repair in BTE, thus the interaction between the hydrogel material and living biological tissue is very important before the applications. Although the biocompatibility of the material was analyzed by cell experiments, proving that the material was non-toxic to cells was a necessary step for the test of biological materials [108]. Models for normal fracture repair (primary and secondary), delayed union, nonunion (atrophic and hypertrophic), segmental defects, and fractures at risk of impaired healing are used in the study of wound healing or fracture treatment [109]. The materials were tested by establishing animal test models, such as rat/mouse, rabbit, dog, sheep, goat, pig, and baboon, to simulate the environmental and physical conditions of the human body.

#### 4.2.1. Bone Defect Model

There are a number of animal models used to assess bone regeneration effects, the most important of which relate to four types of bone defects: calvarial defect, long bone or segmental defect, partial cortical defect, and cancellous bone defect models [110].

##### Calvarial Defect Model

**Figure 8:**
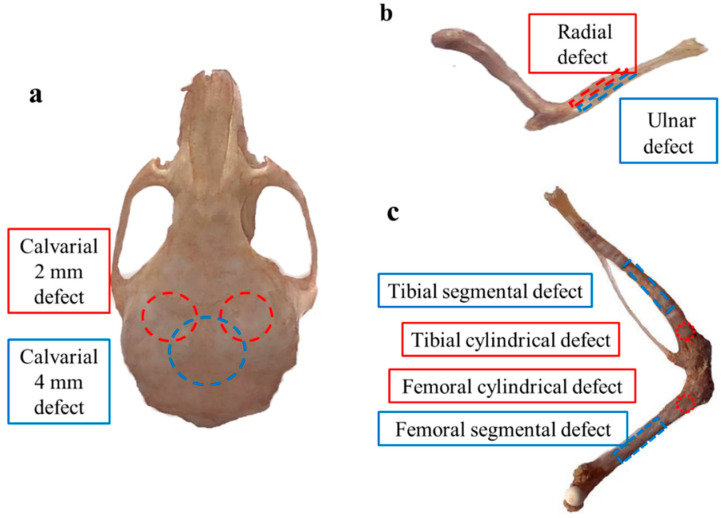
Images of mice calvarial and long bone defect models. (Three models of bone defects: (**a**) calvarial defect model, (**b**) radial or ulnar defect model, (**c**) tibial and femoral defect model).

The calvarial defect (Figure 8a) was produced as follows; a sagittal incision was made on the scalp of the animal and the skin flap was raised to expose calvarial bone, then a standardized circular bone defect was formed by using a trephine bur with saline irrigation to penetrate parietal bone [111]. Finally, reposition the periosteum and suture the closed flap with sutures [112]. Lohmann et al. [113] used the critical-size calvarial bone defect, with an 8 mm diameter defect surgically created in the parietal bone in rats, to evaluate the healing potency of the porous hydrogel. Rodent experimental animals used in the calvarial bone defect model are inexpensive, easy to house, and the bone structure allows for the generation of a standardized defect that can be analyzed using histology and radiographic analysis [114]. The disadvantages of the calvarial defect model are that it is unable to evaluate the performance of materials under physiological mechanical load; the rodent animals have a short lifespan, which is not suitable for long-term research; and the biopsies or blood samples are relatively small [99].

##### Long Bone Defect Model

To overcome these limitations, segmental bone defects in large animals can more closely mimic clinical conditions [111]. The long bone critical-sized defect (Figure 8b,c) is produced by the osteotomy approach, using a drill or saw to remove the desired bone segment from the intended location within the bone. The long bone defect has been modeled in many species [115]. The degree of similarity between the experimental model animal and humans, technical operability, cost, and other factors should be taken into account when the long bone defect is to be modeled in various species. The bone composition of dogs, sheep, goats, and pigs is similar to that of humans, thus it is often used in the research of bone regeneration [116]. Luca et al. [117] evaluated the bone forming ability of rhBMP-2 combined either with chitosan hydrogel or chitosan hydrogel containing β-tricalcium phosphate 15 mm in long bone defects in the radius of a rabbit. Approximately one-third of the length of the tibia, femoral neck, and metatarsal bone is often used to demonstrate segmental injury [118]. In critical-size defect models of ovine tibia, most of the reported defect sizes were 2 to 2.5 times the bone diameter during modeling, and some models with 3 times the bone diameter were used without a clear standard [111]. Large animal long segment bone defect models need to be standardized for wider application. Reasonable choices should be made according to the advantages and disadvantages of different models when evaluating the efficacy of bone repair materials [119].

#### 4.2.2. Fracture Model

There are two fracture animal models of long bone fractures, open osteotomy model and closed fracture model, which play an important role in fracture healing [120].

##### Open Osteotomy Model

The open osteotomy first created a transverse femoral fracture of the femur in the middle, which was then fixed with Kirschner wire [121]. The midshaft femoral closed fracture was closed with a stent after animals received retrograde Kirschner wire fixation in the closed fracture model group [122,123]. The open osteotomy model has a smooth osteotomy surface, and controllable fracture angle and position, but many factors of this model affect fracture healing, such as the combination with soft tissue, periosteum, and even cortical bone damage, as well as certain blood loss and destruction of blood supply at fracture site, resulting in delayed fracture healing [124]. In addition, it takes more time to establish the model, with a higher malunion and nonunion rate [124]. Therefore, compared to the closed fracture model, the open osteotomy model has a significant lag in fracture healing in the early and middle stages, which cannot reflect the true state of fracture healing in this period [124].

##### Closed Fracture Model

It is recommended to select the closed fracture model in the study of fracture healing in the near and middle stages. At present, most of the international models adopt closed fracture models, which have the advantages of simple operation, less damage to soft tissue around the fracture, and less inclusion factors affecting fracture healing [125]. Handool et al. [120] used modified three-point bending pliers, which could make a closed fracture model in rats with minimal postoperative complications, considering the adverse effects and complications of such techniques as osteotomy, drilling the long bones, the use of the guillotine-like apparatus, etc. Bush et al. [5] combines immunomodulatory hemicellulose xylan with chitosan to form a composite hydrogel, and the mouse tibia closed fracture model demonstrates that the hydrogel can promote major remodeling of fracture callus within 4 weeks. Osteoporosis is more likely to result in fractures because of its lower tensile strength [126].

##### Osteoporosis Fractures Model

Ovariectomies and other endocrine procedures in rats and mice, such as orchidectomy, hypovasectomy, and parathyroidectomy, are increasingly being used to study osteoporosis fractures, and older animals are being used to simulate all aspects of osteoporosis in the elderly [126]. Besides, immobilization, dietary changes, and drugs such as steroids have been used in other studies. In the selection of animal models for osteoporosis, it should be noted that the physiological differences of different species should be reasonably selected [126]. Oophorectomy in rats is one of the most commonly used models of osteoporosis, but cortical bone remodeling in this model first appears three months later than adjacent cancellous bone, and Haversian canals only exist in elderly rats, so rats are not a good model of cortical osteoporosis [127].

#### 4.2.3. Others

Many advanced cancers metastasize to the bone, which are characterized by increased osteoclast activity and osteolysis, and are associated with pain, fracture, and nerve compression syndrome, with the end result being a decline in quality of life. Ferreira et al. [128] used female nude Balb/C mice to establish a tumor-bearing mouse model by inoculating 105 MDA-MB-231 cells into the medullar channel of the left tibia of mice. After 15 days of tumor growth, the presence of tumors was confirmed by computed tomography (SPECT) and bioluminescence imaging (BLI). In order to further improve the drug delivery ability of the tumor, PH-sensitive liposome [128] and injectable antitumor slow-release selenium nanocomposite calcium phosphate cement [129] have been applied to bone tumors, and hydrogel is also a potential material.

Infection is one of the most common complications of surgical fracture reduction [130]. Systemic application of prophylactic antibiotics can reduce the incidence of infection. Antibiotic therapy is ineffective due to the vasculature, tissue destruction, and edema, which limit the penetration of antibiotics. These problems can be overcome by using biomaterials to transport antibiotics directly to specific sites of action. Boo et al. [131] used a rabbit humerus osteotomy model, which included osteotomy, plate faxtion, and bacterial inoculum, confirming that gentamicin-loaded hydrogel could successfully prevent infection.

In general, in vivo research design is one of the key links to test the safety and efficacy of biomaterials for BTE, which is one of the most challenging issues [132]. Animal models can be adjusted and modified based on the specific content of the experimental study. Many questions about tissue engineering can be answered with relatively simple small animal models, but the final pre-clinical tests should be performed on large animals to understand if this material is suitable and if it has an optimized conversion from bench to bedside. In future studies, researchers should establish standard operating procedures for animal models to make the results of such studies more comparable and to optimize animal models to develop new treatments [133].

## 5. Prospects

More and more interdisciplinary methods have combined multiple mechanisms to produce hydrogels with enhanced performance, controllability, good biocompatibility, and non-toxicity in BTE. Despite their obvious benefits, hydrogels still lack significant market penetration due to the lack of clinical studies demonstrating their efficacy. Mass production of such materials can be difficult and expensive, so introducing such novel biomaterials into the clinic and market can be challenging [134]. When developing new biomaterials for bone tissue engineering, academic biomaterials scientists often do not consider scale-up and certification-related issues [134].

In fact, many regulations have been established to provide clinical applications and market opportunities for small molecular substances and biological materials. Parathyroid hormone is beneficial to bone healing and has been approved for the treatment of osteoporosis [135]. Teriparatide is an active fragment of parathyroid hormone, which can promote bone anabolism. In December 2002, the Food and Drug Administration approved its listing for the treatment of osteoporosis in postmenopausal women and men [136]. Therefore, many bioactive substances that have osteoinductive effects, such as casein phosphopeptides, steroids, prostaglandin agonists, collagen, and amelogenins, can also be explored in combination with hydrogels. A new type of biomolecule, essential mutant proteins (IDPs), has been recorded to enhance bone cell responses, proving that it is a strong and resilient family of biomolecules [137]. In order to express the best osteogenic potential of IDPs, they have been embedded in a new type of high-performance heterogeneous hybrid bone substitute. It can withstand heavy manufacturing processes (including the use of aggressive solvents and sterilization), regulate delivery methods as needed, and trigger the proliferation and differentiation of bone cells, with a relatively good clinical success record [137]. In the absence of ideal biomedical materials that can be used clinically, efforts should be taken in the direction of composite hydrogels with various growth factors. The final aim is to improve the biological response, thus supporting reconstruction and the formation of new tissue.

On the other hand, we should choose more suitable biological materials and improve the traditional characterization methods. Although hydrogels have been widely studied for their physical and chemical stability, their preparation and application are still difficult due to the complexity of these materials and the organism. It is necessary to select characterization methods more specifically, and to standardize and optimize established animal models to be easier to construct and more similar to human physiological conditions. In addition, factors that affect the safety and stability of hydrogels and osteogenic synthesis factors throughout the production, storage, transportation, treatment, and clinical application of hydrogels should be considered for better clinical application [138].

According to the evaluation results in vivo and in vitro, hydrogel, as a new type of functional polymer material, has been widely used in the field of biomedicine because of its softness, high tissue water content, and good tissue compatibility. At present, some products have been applied in the field of wound dressing, drug sustained-release carrier, and oral protection. All in all, although they are still in development, hydrogels have great potential for future clinical treatment.
